# Wearable Soft Robots: Case Study of Using Shape Memory Alloys in Rehabilitation

**DOI:** 10.3390/bioengineering12030276

**Published:** 2025-03-11

**Authors:** Zain Shami, Tughrul Arslan, Peter Lomax

**Affiliations:** 1 Institute of Micro and Nano Systems (IMNS), School of Engineering, The University of Edinburgh, Edinburgh EH9 3FF, UK; tughrul.arslan@ed.ac.uk (T.A.); peter.lomax@ed.ac.uk (P.L.); 2 Advanced Care Research Center, The University of Edinburgh, Edinburgh EH16 4UX, UK

**Keywords:** shape memory alloys, soft, flexible, robotics, actuation, rehabilitation, wearable, artificial muscle

## Abstract

Shape Memory Alloys (SMAs) have emerged as a promising actuation technology for wearable rehabilitation robots due to their unique properties, including the shape memory effect, high actuation stress, pseudoelasticity, and three-dimensional actuation. With a significantly higher Young’s modulus than biological tissues, SMAs enable efficient and responsive interaction with the human body, making them well suited for musculoskeletal rehabilitation applications. This paper provides a comprehensive review of SMA-based wearable devices for both upper- and lower-limb rehabilitation. It explores their configurations, actuation mechanisms, associated challenges, and optimization strategies to enhance performance. By discussing recent advancements, this review aims to inform researchers and engineers on the development of sustainable, effective, and patient-centric wearable rehabilitation robots.

## 1. Introduction

According to data compiled by the Global Burden of Diseases (GBD), about 1.71 billion people suffer from musculoskeletal disorders. According to the study, low back pain is the main contributor to these musculoskeletal conditions. Other contributors include osteoarthritis, fractures, other injuries, amputations, neck pain, and rheumatoid arthritis [1]. Although the effects of musculoskeletal conditions are more apparent in the older population, younger people can also experience them, especially during their peak income-earning years. Musculoskeletal conditions contribute the most to years lived with disability (YLDs) worldwide with approximately 149 million YLDs, which accounts for 17% of all YLDs worldwide [2].

Wearables are being used to provide support for rehabilitation therapy in hospitals and rehabilitation centers. Many of these devices are actuated by DC motors but are static with varying degrees of freedom (DOF). They need to operate in special environments and are not suitable for use in daily life. If devices could be developed for daily use, they would provide not only the autonomy a person needs but also the musculoskeletal rehabilitation they require. Patients have suggested that the devices they require should be easy to use, safe, durable, less distinctive, custom-made, affordable, small, and lightweight [3]. These requirements provide a foundation for an optimized exoskeleton.

**Figure 1:**
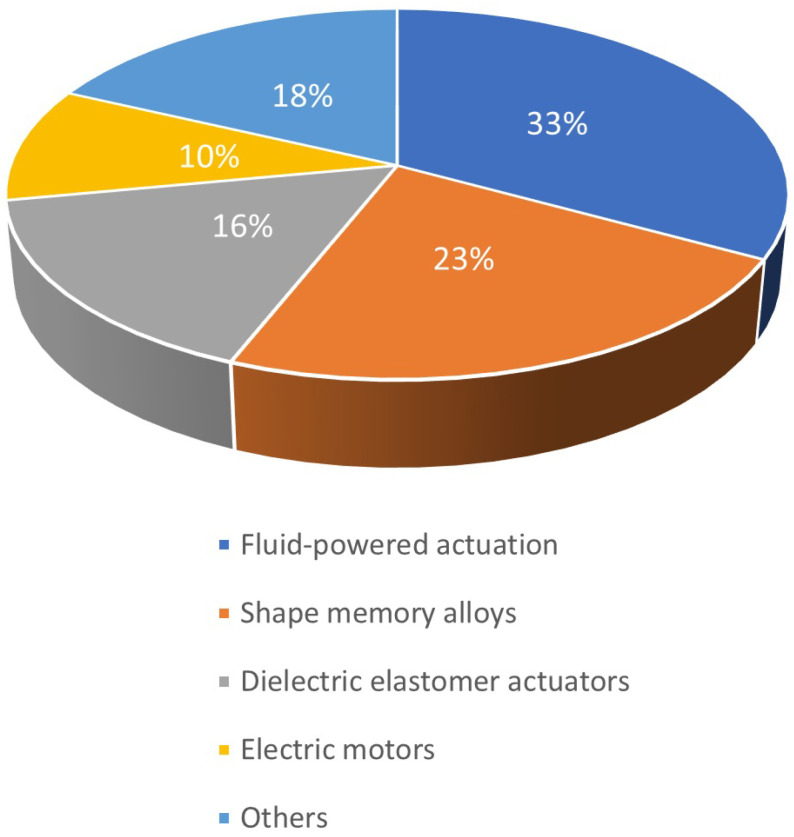
The percentage of the number of research articles with different actuation mechanisms applied in soft robotic devices for rehabilitation as of November 2021 [7].

Soft materials are materials that have properties similar to those of living tissues [4]. This class of materials and devices made from them is generically referred to as artificial muscle. These materials and devices can reversibly contract, expand, and rotate [5] due to an external stimulus (e.g., current, voltage, temperature, pressure, etc.) [6]. Shape Memory Alloys and dielectric elastomers are examples of artificial muscle. Figure 1 shows the percentages of different actuation mechanisms investigated for use in robotic rehabilitative devices. Shape Memory Alloys are the second most studied actuation mechanism, having been investigated in 23% of the research articles concerning soft robotic devices for rehabilitation [7].

In recent years, exoskeletons have been actuated using different actuation mechanisms: AC and DC motors, fluid-powered actuators (hydraulic and pneumatic), dielectric elastomers, and Shape Memory Alloys [8]. Currently, electric motors are the preferred actuation mechanism in exoskeletons, but they can cause considerable limitations to their operation due to their excessive weight and operation noise. Fluid-powered actuators (pneumatic and hydraulic) that provide a decent force-to-weight relationship are again limited in their application by the need for compressed air and a fluid tank [6] as well as operation noise. The use of Shape Memory Alloy actuators may reduce weight and noise, but they still require complex electronic control systems to function. Recent advances in electronics have made the development of such control systems relatively simpler.

In the rapidly evolving field of rehabilitation robotics, it is essential to compare SMA-based devices with other emerging technologies such as dielectric elastomer actuators (DEAs) and piezoelectric materials. While DEAs offer high energy density and large strain capabilities, SMAs provide distinct advantages in terms of simplicity of control and energy efficiency [9]. For instance, SMAs can achieve significant actuation with minimal energy input, making them suitable for applications where battery life is a concern [10].

Moreover, the scalability of SMA technology is a crucial factor in its favor. Unlike piezoelectric materials, which may require complex circuitry and control systems, SMA actuators can be integrated into simpler designs, facilitating easier implementation in wearable devices [11].

### 1.1. Research Gap

While the potential of SMAs is acknowledged, it is essential to clarify the unmet needs within rehabilitation robotics that these materials can address. Current rehabilitation devices often struggle with limitations such as bulkiness, energy inefficiency, and inadequate adaptability to user needs. SMAs, with their unique properties of thermal shape memory effect and pseudoelasticity, can provide lightweight, compact solutions that enhance user comfort and mobility [12]. For instance, the ability of SMAs to undergo significant deformation while maintaining a high force-to-weight ratio allows for the design of more ergonomic exoskeletons that can better conform to the human body [13]. This adaptability is particularly crucial for patients with varying degrees of mobility impairment, where traditional rigid actuators may not provide the necessary flexibility and responsiveness [14].

Moreover, the integration of SMAs in soft robotics can lead to devices that are not only lighter but also more compliant, allowing for safer interactions with users [15]. This compliance is vital in rehabilitation scenarios where the risk of injury from rigid devices is a concern. By addressing these specific gaps, this review highlights how SMAs can revolutionize the design and functionality of rehabilitation robots, moving beyond a mere summary of existing studies to a focused discussion on innovative applications and potential advancements.

### 1.2. Novelty

Although SMA-based devices for assistance and neuromuscular rehabilitation have been previously discussed [7,16,17,18,19,20,21,22], this paper concentrates on joint movement assistive devices with an emphasis on SMA-based actuation. The novel aspects of this paper are as follows:

- It discusses SMA-based devices for the upper limb and lower limb that are in the prototype phase. It also discusses the challenges that these devices are facing to provide insight into developing a more effective SMA-based rehabilitation device.
- It elaborates on the design standards of joint movement assistive devices, covering a range of fields including (but not limited to) SMA configuration, actuation techniques, and cooling mechanisms.
- In addition to conventional exoskeletons, it discusses SMA-based smart fabrics, which are the state of the art in the field.
- This paper discusses efforts to implement the physical human–robotic interaction (PHRI) in the design of wearables for rehabilitation.

## 2. Shape Memory Effect and Pseudoelasticity

**Figure 2:**
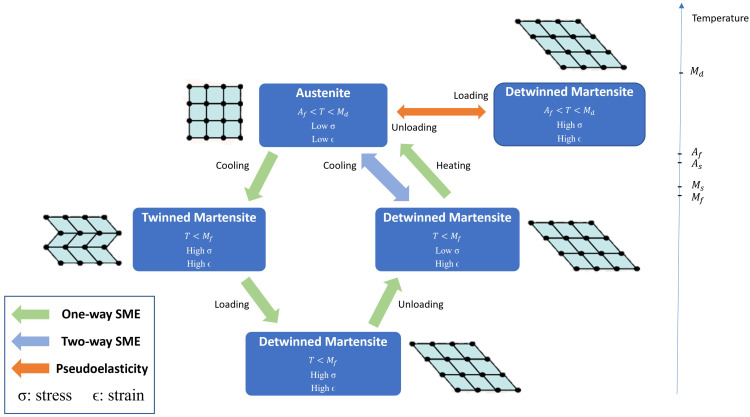
Phase transformation process, adapted from [24].

The shape memory effect (SME) is the phenomenon whereby a material is given a temporary shape to recover its original shape on being externally stimulated. The SME was first discovered in 1932 in the Gold–Cadmium (Au-Cd) alloy [23]. Buehler and Wang are credited with the discovery of SME behavior in the famous alloy of Nickel and Titanium that they named ‘Nitinol’ (Nickel–Titanium Naval Ordinance Labs) after the laboratory for which they worked. Nitinol is the most studied and popular [24,25] alloy showing the SME due to its higher stability and thermomechanical performance [26]. The metallic alloys that demonstrate SME behavior are called Shape Memory Alloys (SMAs). The SME in these alloys occurs by a reversible phase transition upon variation in temperature. In this phase transition process, the SMA possesses three different crystalline structures at each stage, twinned martensite, detwinned martensite, and austenite [27]. In a one-way shape memory process (OWSMP), the SMA starts as twinned martensite at a low temperature, which transforms into detwinned martensite with deformation upon mechanical loading. When heated to the austenite start temperature ($A_{s}$), it begins to transform to the austenite phase (recovering its original shape). The transformation is completed at the austenite finish temperature ($A_{f}$). During this process, the nitinol wire is able to contract to a maximum of 4.5% of its length with Poisson’s ratio 0.33 [28]. Upon cooling to the martensite start temperature ($M_{s}$), the SMA starts to return to the twinned martensite phase. The phase transformation is complete at the martensite finish temperature ($M_{f}$). The maximum temperature at which martensite can be stress-induced is $M_{d}$ [29]. After $M_{d}$, the SMA deforms permanently, like any other metal [24]. In the two-way shape memory process (TWSMP), the SMA remembers its shape at both high and low temperatures. Such SMAs are not commercially viable as they require training and can recover only half the strain compared to the OWSMP for the same material [30,31,32], and this strain tends to deteriorate rapidly, particularly at higher temperatures [33]. Therefore, OWSMP is a more economical and reliable solution. Numerous training methods have been proposed, two of which are external and spontaneous load-assisted induction. The conductive SMA is usually transformed through the Joule Heating Effect (passing a current through an electrical conductor to produce thermal energy) [28]. Pseudoelasticity (PE) or superelasticity (SE) is the phenomenon where upon the application of a mechanical load at a temperature between $A_{f}$ and $M_{d}$, the SMA reverts to its original shape without thermal activation [34]. Figure 2 shows the shape memory transformation process.

## 3. Shape Memory Alloy-Based Actuators

### 3.1. Design

SMAs have attracted attention to be used as artificial muscles due to their soft nature. SMA actuators have a good force-to-weight ratio, a small volume, and a noiseless operation [22]. Therefore, SMA actuators are a good alternative for actuation in wearable and soft robotic applications, particularly rehabilitation devices. SMAs in the form of wires can achieve a displacement of 3–5%, whereas in the form of springs they can generate large active displacements (>100%) while maintaining significant forces [35,36].

NiTi alloys have been used in actuators in different shapes, such as wires, beams, rods, tubes, films, cables, and springs; the most common forms are still wires and springs [15]. Different SMA-based actuators use different configurations of these wires to achieve the desired function. The main factors determining the actuation speed and frequency of an SMA actuator are power and cooling time. The force provided by the actuator depends on the diameter of the wire and the number of wires. Properties of NiTi wires commonly used in these actuators are given in Table 1.

Although a larger wire diameter produces a larger heating pull force, a higher current is needed for it to start contracting and a longer cooling time is required [38]. Thus, when designing an SMA actuator, these trade-offs must be considered.

While nickel–titanium (NiTi) alloys are the most commonly used SMAs, there are other materials that show promise for rehabilitation applications. For instance, copper-based SMAs have been explored for their potential advantages, including lower cost and improved thermal properties [15]. However, challenges such as lower fatigue resistance and limited operational temperature ranges must be addressed before these materials can be widely adopted in rehabilitation devices [39].

### 3.2. Environmental Influences

The performance of SMAs is significantly influenced by environmental factors, particularly temperature and humidity, which can affect phase transition temperatures (*A_s_*, *A_f_*, *M_s_*, *M_f_*) and the overall shape memory effect. Variations in humidity can lead to changes in the mechanical properties of SMAs, impacting their actuation performance [15]. For example, increased humidity can lower the transition temperatures, potentially leading to premature activation or reduced efficiency in actuation cycles [40]. This variability necessitates a robust design approach that considers environmental conditions to ensure consistent performance across different settings.

Furthermore, the manufacturing process of SMAs can introduce variations that affect their phase transition characteristics. Factors such as alloy composition, processing techniques, and heat treatment can lead to discrepancies in the expected performance of SMA actuators [39]. Understanding these manufacturing variations is crucial for developing reliable SMA-based devices that can perform consistently in real-world rehabilitation scenarios.

### 3.3. Advantages

There are many advantages to using SMA actuators. One of which is that compared to electric motors, hydraulics, and pneumatics, SMAs have unique properties (such as high energy density, biocompatibility, and wear resistance) and can respond directly to stimuli from the environment. Thus, actuators developed using SMAs are cheaper, less mechanically complex, and smaller. Nitinol shows the highest work density at 10 J ${\mathrm{cm}}^{3}$ (a factor 25 times greater than that for electric motors). It can lift an object more than a hundred times its weight. Nitinol is very suitable for designing actuators where large displacements and forces are required but a quick response time and high efficiency are not. Due to the flexible nature of SMAs, SMA-based actuators can provide actuation in any direction [24].

SMA actuators offer silent operation, high energy density, and increased flexibility in design. SMA wire actuators allow lightweight and clean operation without any noise or hindrance in motion due to the absence of friction and moving parts. SMA actuators can work under various deformation modes such as rotation, compression, tension, bending, and their combinations [41].

#### 3.3.1. High Energy Density

The claim that SMAs possess a high energy density is well supported in the literature, particularly when compared to traditional electric motors and fluid-powered actuators. Research indicates that SMAs can achieve energy densities significantly higher than those of conventional actuators, making them particularly suitable for applications where weight and space are critical constraints [42]. For example, SMA actuators can provide energy densities in the range of 5–10 times greater than that of typical electric motors, which is a substantial advantage in the context of wearable rehabilitation devices [11].

This comparison of energy density not only highlights the potential of SMAs to enhance the performance of rehabilitation devices but also emphasizes the importance of integrating these materials into designs that prioritize efficiency and user comfort.

#### 3.3.2. Pseudoelasticity

Pseudoelasticity, a characteristic of certain SMAs, plays a vital role in enhancing the functionality and performance of rehabilitation devices. Unlike traditional materials, pseudoelastic SMAs can undergo large strains without permanent deformation, making them ideal for applications requiring dynamic movement and adaptability [13]. This property is particularly beneficial in rehabilitation scenarios where users may experience varying levels of force and movement, allowing the device to respond effectively to the user’s needs without compromising structural integrity.

The advantages of using pseudoelastic SMAs over non-pseudoelastic ones include improved energy efficiency and reduced weight, which are critical factors in the design of wearable devices [43]. For example, the ability of pseudoelastic SMAs to return to their original shape after deformation allows for the development of lighter and more compact actuators that can operate effectively in rehabilitation settings [44].

#### 3.3.3. Cost-Effectiveness of SMA-Based Devices

Cost-effectiveness is a critical consideration in the development of rehabilitation devices. SMAs, while offering unique advantages, must also compete with traditional actuation technologies in terms of affordability and accessibility. Research indicates that SMA-based devices can be more cost-effective than their electric motor counterparts due to lower material costs and reduced complexity in design [42]. For instance, the compact nature of SMA actuators allows for simpler mechanical designs, which can lower manufacturing costs and enhance scalability [11].

Additionally, the energy efficiency of SMA actuators can lead to reduced operational costs over time, particularly in applications requiring prolonged use, such as rehabilitation therapy [45].

### 3.4. Challenges and Suggested Solutions

The three major challenges of using SMA actuators are that the SMAs experience a reduced strain recovery, have a low working frequency, and have low energy efficiency (10–15%) and behavior under cyclic loading.

#### 3.4.1. Hysteresis

Hysteresis is the difference measured between the heating and cooling transition temperatures ($\Delta T=A_{f}-M_{s}$), which is defined between the temperatures where the state of the material is 50% austenite while heating and 50% martensite while cooling [46]. This property is essential when selecting the most suitable SMA material for the desired application, e.g., fast actuation requires a small hysteresis, and retaining the shape for a large temperature range requires a large hysteresis [47]. A hysteresis effect occurs in the SMA wires due to sequential loading and unloading, reducing their strain recovery due to plastic strain accumulation. In a recent study, an SMA wire with a diameter of 1.5 mm and 560 mm in length was subjected to 1000 cycles and showed a 3 mm residual deformation or 0.5% residual strain. This residual strain was eliminated by applying a 1.7% pre-strain on the SMA wire [48]. Therefore, the pre-strained SMA wires become impartial to the hysteresis effect.

#### 3.4.2. Work Frequency

The low working frequency is attributed to a sluggish cooling process caused by a low heat transfer rate. To address this limitation, various approaches have been explored. Among them, reducing the diameter of the SMA fiber to enhance the surface area has emerged as the most effective strategy. Techniques to increase heat convection have also been investigated. Some of the techniques investigated include the use of forced air convection, immersion of the actuator in fluid, use of thermally conductive pastes [49], and using heat sinks [50,51]. During the investigation of cooling times and contraction of SMA wires under the influence of air cooling, it was discovered that employing mechanical means to move air across the actuator resulted in a significant enhancement in the convective heat transfer coefficient. Specifically, it was observed that the convective heat transfer coefficient could be increased by a factor of 8 [52]. Water or ethanol cooling and nucleate boiling were observed to greatly enhance the cooling rate, allowing a response time of a few milliseconds [53]. While immersing the SMA actuator in fluid may greatly enhance the cooling rate, it increases the power required to heat the wire. An example of this is given by the increase in the power consumption of the crab-inspired robot, from 1 kW in air to 10 kW in water [54]. Likewise, a technique used silicone tubing to encase the springs to elevate their actuation frequency. By passing air and water through the tubing, the convective cooling rate was enhanced. As a result of this technique, an intracranial robot achieved an actuation frequency of 0.143 Hz [55]. To further enhance the actuation frequency, an alternative method involved submerging an SMA wire in a conductive paste contained within a conforming shell. Similar to the water-immersion technique, the power consumption increases as the cooling time decreases. The reduction in cooling time is also regulated by the dimensions of the compliant shell and the selection of the conductive paste [56]. To enhance cooling efficiency, heat sinks have been used to extract heat from a cooling SMA actuator. In the development of an antagonistic SMA wire actuator, a mobile heat sink was introduced, establishing contact with the cooling SMA wire through the force exerted on it by a heated SMA wire. Although this approach has the potential to elevate actuation frequency, it also introduces greater complexity to the actuator [57].

#### 3.4.3. Energy Efficiency

SMAs have low energy efficiency as they require more thermal energy than the mechanical output they provide. Many strategies have been suggested to improve the energy efficiency of these alloys. One such strategy involves using short high-voltage pulses instead of the traditional quasi-static activation process based on low voltage. This strategy proposed using simple hardware to provide short voltage impulses for a short period of time instead of the expensive and complex electronics used for current control. It achieved high actuation speeds with low consumption of energy. The technique was able to provide close to 10% stroke actuation in the range of 40 ms. This is because, with short activation pulses, there is no energy loss to the environment. For a 76 µm wire, adiabatic heating was achieved at a voltage of around 24 V. This process achieved energy conservation of up to 80% [38]. An SMA-based actuator design investigated to increase energy efficiency is the bi-stable element design concept. The bi-stable element is a thin beam of sheet metal. The pivotal mounting of the element is a crucial aspect of this design. In this particular arrangement, the element is mounted on two pivot joints, with the SMA wire connected on each side of a joint. This configuration allows the bi-stable element to switch positions whenever one of the two SMA wires is activated. The closer the SMA wires are to the pivot point, the smaller the actuation stroke needed from the SMA wire to induce the snapping effect, but a larger force would be required from the SMA wire. This force can be increased using a wire of a thicker diameter or by adding more wires in a parallel configuration. This transmission mechanism can provide larger strokes by utilizing the high energy density of the SMA wire. It allows for the design of an actuation system with high activation frequency, high performance, small design space, and high stroke [58].

#### 3.4.4. Fatigue Property

For rehabilitation applications, considering the fatigue characteristic of Shape Memory Alloys (SMAs) is crucial when they are applied practically. Fatigue refers to the gradual deterioration of material properties due to repetitive or cyclic loading. Gaining insights into the fatigue behavior of SMA actuators is vital to ensuring their long-term reliability and performance. In SMAs, three distinct types of fatigue are of notable importance. Firstly, there is the conventional fatigue leading to failure due to fracture, which arises from mechanical cycling under stress or strain at a constant temperature. Secondly, there are alterations in material properties, such as transformation temperature and transformation hysteresis, induced by thermal cycling during the transformation process. Lastly, there is the degradation of the shape memory effect (SME) caused by either mechanical or thermal cycling [59].

SMA materials exhibit unique fatigue characteristics due to their phase transformation behavior. The cyclic loading experienced by SMA actuators during repeated actuation can induce stress accumulation, resulting in fatigue damage and eventual failure. Several factors contribute to the fatigue behavior of SMA actuators, including actuator design, material composition, loading conditions, and operating temperature.

Various testing methodologies such as cyclic loading tests [60,61,62,63,64], strain-life tests [65,66,67,68], and stress-life tests [69,70,71] are used to assess the fatigue performance of SMA actuators. These tests involve subjecting the SMA actuator to repetitive loading cycles while monitoring the actuator’s response and measuring parameters such as strain, stress, or displacement. By analyzing the data obtained, researchers can evaluate the fatigue life of the actuator, the endurance limit, and the fatigue strength.

Studies have shown that the fatigue behavior of SMA actuators can vary significantly depending on the specific alloy composition [65,72,73], manufacturing process [73,74,75], and the thermomechanical treatment [76,77,78]. For example, the fatigue properties of NiTi-based SMA actuators were investigated, and it was observed that fatigue life and endurance limit were influenced by factors such as loading frequency, stress amplitude, and thermal cycling [79].

Furthermore, researchers have explored different techniques to enhance the fatigue resistance of SMA actuators. These techniques include alloy composition optimization, surface treatment, and mechanical strengthening. For instance, the effect of surface modification on the fatigue behavior of SMA actuators was investigated, and it demonstrated improved fatigue life through shot peening treatment [80].

To ensure the reliability and durability of SMA actuators in rehabilitation applications, it is crucial to understand their fatigue behavior and develop strategies to mitigate fatigue-related failures. By conducting rigorous fatigue testing, optimizing material properties, and employing appropriate design practices, the fatigue performance of SMA actuators can be improved, leading to more reliable and long-lasting rehabilitation devices.

### 3.5. Influencing Factors for Design of Variables

**Figure 3:**
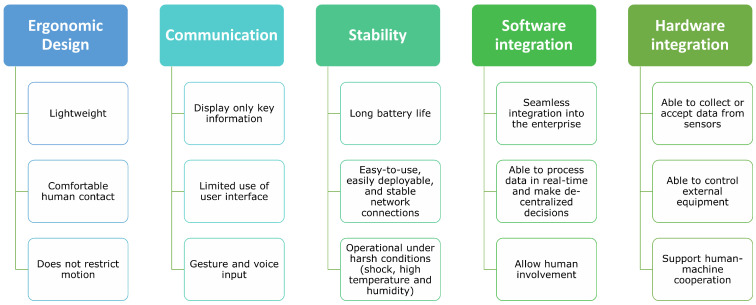
Factors influencing design of wearables [81,82].

Based on the feedback from users of assistive wearable devices, five critical factors are highlighted in Figure 3.

#### 3.5.1. Physical Human–Robot Interaction (pHRI)

Wearable robots of all types should be comfortable, safe, and able to interact with the user smoothly. Comfort and safety in an exoskeleton are influenced by actuation, control, and the kinematics of structural design. There has been significant work in designing novel actuators for wearable robots that significantly improve safety during human–machine interaction through compliant actuators and advanced control systems. One area that still needs attention is the kinematic and mechanical design of wearables to achieve optimal physical robot–human interaction (pHRI) [81].

It is very challenging to design the physical coupling between the wearable robot and the human user. The commonly used approach of fastening the wearable to the body while having a soft interface material is not sufficient. It makes it a challenging task to simultaneously improve comfort and attach the robot to the body at the physical human–robot interaction interface (pHRII). As a result of the soft interaction between human tissue and pHRII materials, the sensors at the interface become incapable of describing the behavior experimentally. To improve the design of a wearable, it is crucial to model the interaction between the limb and the wearable [83].

Presently, an overall pHRII model does not exist, but previous studies have addressed some issues pertaining to its design and robot control [83]. The approach initially applied was centered around ensuring joint axes’ concentricity and passive realignment by ensuring extra degrees of freedom [84]. It was shown that applying optimization techniques on redundant joint angles measured allows for the estimation of unknown positions of the human for effective control [85]. In order to effectively transfer the force across the leg socket so that discomfort and tissue damage are minimized, the stiffness map of the socket needs to be tuned for equal distribution of pressure across all points [86]. Inverting the stiffness profile of a human hip was found be beneficial in designing the attachment, which was in contact with superficial skeletal structures rather than softer tissue [87]. Designing a pHRII is a field that has only recently been explored and found to be an iterative, prototype-based process. To ensure maximum effectiveness, designers iterate by manipulating parameters such as device pHRII compliance and use features such as human–robot positional errors. This approach has been found to be time-consuming, difficult to standardize, and expensive.

To establish a standardized approach for designing pHRII, it is important to characterize the human–robot interaction. To achieve this, a novel simulation-based model of the pHRII was developed using MATLAB 2017b (MathWorks Inc., Natick, MA, USA). This model was named HuRoSim. It was capable of simulating viscoelastic properties of human skin and soft tissue, as well as interface material and any medium of attachment. HuRoSim focused on the relative movement between the human and the robot while considering the reaction forces at the point of contact (pHRII) due to the loads applied across it. Using this approach, optimal design parameters such as geometry, strap stiffness, compliance, pretension, etc., could be computed. It also allows for robot configuration analysis and monitoring of the effects of variations in human size and tissue properties [83].

#### 3.5.2. Control Mechanism

To achieve efficient and precise rehabilitation results, the control of the SMA actuators plays a very crucial role. Various control strategies have been developed to address the challenges associated with the actuation of SMAs, including stress-based control [88,89], temperature-based control [90,91], and hybrid control [92,93].

The most common method used for SMA actuators is temperature-based control. By manipulating the temperature of the SMA, the actuator can be cycled between its two distinct phases: austenite and martensite. The transformation between these phases leads to a change in the actuator’s shape. Temperature-based control involves heating the SMA above its transformation temperature to induce the austenite phase and cooling it below the transformation temperature to trigger the martensite phase. Precise temperature control is essential to achieving accurate actuation and preventing overheating or thermal damage to surrounding tissues.

Stress-based control is another approach used for SMA actuators in rehabilitation. External stress is applied to the actuator in this method, which induces a transformation in the SMA material. Stress can be generated by external devices, such as springs, hydraulic systems, or mechanical links. By carefully controlling the applied stress, the actuator can be modulated to achieve the desired shape change. Stress-based control offers advantages such as simplicity and independence from temperature fluctuations, but it requires careful calibration and monitoring to ensure the actuator operates within its safe limits.

Hybrid control approaches combine both temperature and stress-based control strategies to enhance the performance of SMA actuators. These methods take advantage of both approaches while mitigating their individual limitations. By integrating temperature and stress feedback systems, hybrid control allows for more accurate and robust control of SMA actuators in rehabilitation applications.

In recent years, various control algorithms and techniques have been developed to improve the control of SMA actuators in rehabilitation. These include model-based control methods, such as proportional-integrative-derivative (PID) control, fuzzy logic control, and adaptive control [94,95,96]. These algorithms aim to optimize the actuator’s response, improve accuracy, and enhance the adaptability to different patient needs.

The integration of energy efficiency strategies, such as the short high-voltage pulse strategy, into existing control systems for rehabilitation robots is a critical area for exploration. This strategy allows for precise control of SMA actuators, enabling them to operate effectively while minimizing energy consumption [11]. By employing advanced control algorithms, such as PID controllers, researchers have demonstrated that SMA actuators can achieve high levels of accuracy and responsiveness, which are essential for rehabilitation applications [43].

Furthermore, the development of adaptive control systems that can dynamically adjust to user movements and environmental conditions will enhance the energy efficiency of SMA-based devices.

## 4. Applications of SMAs in Exoskeletons

Due to the their low complexity, high force-to-weight ratio, high flexibility, silent operation, and high energy density, SMAs have had many reported applications in exoskeletons, both for the upper and lower limbs. This section discusses some of these applications with an emphasis on the following:

- How SMAs have featured in the design of the device.
- The target organ and the intended motion achieved.
- The challenges encountered during the use of these devices.

### 4.1. Upper Limb

**Figure 4:**
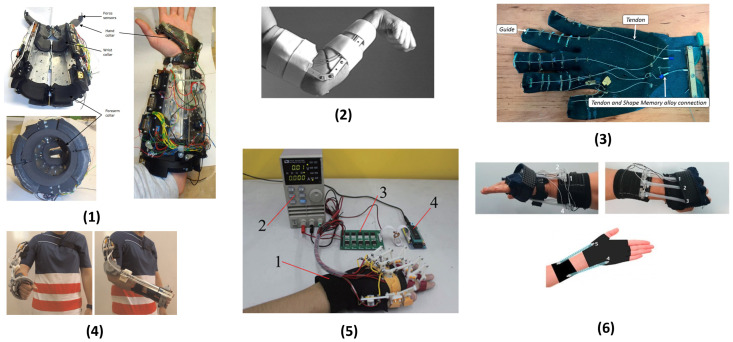
(**1**) Forearm and hand rehabilitation exoskeleton with 3 DOF [97], (**2**) EDGES SMA-based actuator [98], (**3**) Advanced Service Laboratories (ASR) glove for hand rehabilitation [99], (**4**) Bowden cable-based SMA actuator [100], (**5**) four-bar link mechanism-based SMA finger actuator: 1. Hand exoskeleton, 2. Constant current source, 3. Drive circuit, 4. Control circuit [101], (**6**) tube-encased SMA coil wrist actuator [102].

The relevant literature that reported SMA-based actuation techniques used in upper-limb exoskeletons has been discussed briefly in this section and presented in Table 2 and Figure 4, which displays the target organ, target motion, actuation mechanism, cooling mechanism, and challenges.

#### 4.1.1. Forearm and Hand Rehabilitation Exoskeleton with 3 Degrees of Freedom (DOF)

A wearable prototype exoskeleton was designed having three degrees of freedom to provide rehabilitation therapy at home. The actuation modules for the prototype have a differential configuration and consist of SMA wires, position sensors, a bias spring, and a force amplification mechanism.

Two 0.2 mm diameter SMA wires were used in a parallel configuration because their minimum bend radius, 20 mm, was similar in size to the module componentry. The exoskeleton has three coupling points, the hand collar, wrist collar, and forearm collar. It can be split into two parts for easy doffing and donning. Flexion–extension and abduction–adduction are controlled by the variation in the length of four cables (called tendons) through a spring bias configuration. The tendons are arranged such that there is one pair above the wrist (on either side of the center of rotation) and one pair below the wrist. This is performed to allow for complex combinations of movements. Each tendon is independently controlled because it is connected to an actuator and its mechanics are housed in a separate module. It is considered soft robotic, as the tendons (having flexible cables) span this joint. Pronation and supination are controlled by two tendons that wrap around the forearm collar circumference through a differential configuration. The two tendons work antagonistically to provide the two movements. The lengths of all the tendons are controlled by the displacement of their respective SMA actuators. This displacement is measured by rotary potentiometers. Force sensors, arranged circumferentially around the hand, measure the torques for the flexion–extension and abduction–adduction.

The control system was able to track sinusoidal trajectories and worked on the concept of regulating stiffness in a differential configuration. To decrease the response time and increase the cooling rate, miniature fans were used [97].

#### 4.1.2. EDGES SMA-Based Actuator

The application of Nickel–Titanium (NiTi) in rehabilitative exoskeletons due its superelastic nature was initially investigated in a compliant brace (EDGES) to promote spastic elbow relaxation. It used NiTi’s pseudoelasticity to supplement elbow movement without constraining its natural movement. A commercial NiTi alloy (heat-treated at 400 °C for 1 h and Water-Quenched) was used to provide the actuation to the exoskeleton. The prototype was assembled using two thermoplastic shells that were connected by polycentric hinges. The actuation was provided by four 2 mm diameter NiTi bars that were housed in the upper-arm shell and slid through the cylindrical fixture of the forearm.

The wires were trained through cyclic loading to eliminate residual strain. Through bending tests, it was found that the moment provided by the exoskeleton increased with angles between 20 and 80°, after which it reached a peak torque at 0.95 Nm for a two-wire configuration and 1.9 Nm for a four-wire configuration. These figures are lower than the maximal muscle flexor and passive-reflex torques. This shows that the actuator can stretch the muscles without fixing the position of the elbow, during both involuntary jerks and voluntary movement.

The control system was open-loop; the NiTi provided the corrective torque, which was measured using optoelectronics. The measuring protocol used four infrared (IR) cameras to track the angle of the elbow through three IR markers (placed on the shoulder, elbow, and forearm) [98].

#### 4.1.3. Advanced Service Laboratories (ASR) Glove for Hand Rehabilitation

The design of the wearable hand robot consists of a glove (main body), several guides (connect tendons), tendons (mediums for actuation in two degrees of freedom) and SMA actuators (provide actuation). Each of the tendons are fastened at their two ends at the proximal and distant phalanx. Each tendon is connected to an SMA actuator at one of its ends, and the SMA actuator is connected to a point in the platform mounted on the forearm. When each SMA is actuated, the length variation and tension force are transmitted to the associated tendon, generating the motion of a phalanx. The distal tendon is used for moving both the proximal interphalangeal (PIP) joint and distal interphalangeal (DIP), while the proximal is utilized for the metacarpophalangeal (MCP) joint. The SMA wires were 0.38 mm in diameter and were actuated through a 2.2 A current. Air fans in the actuator module were used to cool the SMA wire. Each fingertip could generate a 10 N force, and the robot could provide a 40 N gripping force, which is sufficient for ADLs [99].

#### 4.1.4. Bowden Cable-Based SMA Actuator

The actuator consisted of one or more SMA wires, a polytetrafluorethylene (PTFE) tube, a Bowden cable, and the terminal parts.

A Bowden cable, comprising a nylon sheath-covered metallic spiral, was used to transmit force. This cable has the advantage of being flexible and helps in heat dissipation during the cooling stage of the SMA wire [40]. A Bowden cable of 3.5 mm diameter was used for one SMA wire, but a cable of 6.5 mm was used for up to five SMA wires with a diameter of 0.51 mm. The PTFE tube facilitates the SMA wires’ displacement and provides electrical insulation between the SMA wires themselves as well as the SMA wire and the Bowden tube. It has the advantage of being capable of working at temperatures higher than 250 °C as well as being a transparent, chemically inert, and nontoxic material.

The SMA wire provides the actuation by contracting via the Joule Heating Effect. The force and final displacement requirements of the device dictate what diameter and length of wire are used as well as the number of wires used. The terminal unit fixes the Bowden cable with the SMA wire, at one end, and the actuated system or the tendons of the actuated system with the SMA wire, at the other end. It consists of two pieces screwed together to permit tension in the SMA wire after it has been mounted for the final application. They are also the connectors for supplying power to the actuator.

The flexibility and easy integration of this SMA actuator make it very suitable for musculoskeletal rehabilitation because the actuator would no longer restrict joints but supplement their movement, thus giving the name ‘soft robotics’ to this approach.

Due to the hysteresis effect of the SMA wire, a four-term bilinear proportional-integrative-derivative (BPID) controller was used to control the actuator where a PWM (Pulse Width Modulation) signal (modulated by the error signal) was used to regulate the current supply. The response was provided by a position sensor, which was then compared with the desired reference.

This proposed control algorithm was seen to provide long-term stability and durability to the actuator, but the only problem lay in the cooling time for the SMA wire to return to the Martensite phase, which limited the actuator speed [100].

#### 4.1.5. Four-Bar Link Mechanism-Based SMA Finger Actuator

An SMA-based actuator was designed for an exoskeleton for hand rehabilitation. The actuator used two four-bar linkages and a gear assembly to provide the actuation. The length of the link affects the performance of the exoskeleton. Therefore, the length was kept reasonable to increase the efficiency and decrease torque resistance.

The design of the exoskeleton was focused on the physiological structure of the finger. The structure comprises the proximal, middle, and distal phalanges. The connection of the phalanges is in the sequence of MCP, proximal PIP, and DIP joints. MCP, PIP, and DIP joints are responsible for the flexion and extension, whereas the adduction and abduction are made possible through the MCP joint. The PIP joint rotation speed is 3–4 times the rotation speed of the MCP joint.

The constraints applied to the structure were as follows:

- The operation would cover the entire range of finger motion.
- The rotation speed ratio of the PIP and MCP joint would be between 3 and 4.
- The input torque would be as small as possible.
- To satisfy all the constraints, lengths were calculated through kinematic analysis for a gear ratio of 2:1 of the proposed structure.

A pair of SMA wire springs (SMA-I and SMA-II) rotate the drive link (R1), which moves the whole exoskeleton. This happens in three steps:

1. The fingers are initially kept straight.
2. SMA-I automatically contracts when heated, forcing R1 to rotate clockwise.
3. When SMA-II is heated, it contracts, rotating R1 anticlockwise to return the exoskeleton back to its initial state.

The contraction or stretch length of the SMA spring was 10 mm, and it could provide a force of 20 N. A current of 2 A was used to energize the SMA wire of 0.5 mm diameter, taking 3 s to reach a temperature of 80 °C to achieve the phase transformation, martensite to austenite.

The exoskeleton was controlled by turning the heating current on and off in the 10 SMA springs to achieve the specified rehabilitation movement precisely by considering parameters, e.g., heating order, response speed, and heating time [101].

#### 4.1.6. Tube-Encased SMA Coil Wrist Actuator

An SMA-based soft wearable robot was designed to assist wrist motion in patients with a difficulty in manipulating their lower arm. The SMA was shaped in the form of a spring to provide a substantially larger displacement. Coil-shaped SMA spring actuators were found suitable for this application as they experience an extension strain of over 200%, greater than the strain shown by human muscles. The actuator consists of one SMA coil spring surrounded by a stretchy polymer (Ecoflex 00-30) tube. A coolant circulation system was added to the actuator to cool the SMA spring. A current was provided through electric wires at both ends of the SMA spring, which was fixed to the polycarbonate connectors that contained the flow inlet and outlet [102]. To investigate the most suitable spring parameters for optimum performance, five sample springs were prepared and investigated [103,104]. The force required was 10 N per coil spring. The maximum target contraction ratio was kept similar to the human muscle, i.e., release above 40% of its initial stretched length. Upon application of a 10 N load, the targeted displacement range that was selected was 50 mm from the initial stretched length. To cater to the wrist, the targeted maximum length of the spring was chosen to be 150 mm. The considerations for the actuator were that it was kept thin to make it as portable and easy to wear as possible and the maximum surface temperature was kept as low as possible to prevent the burning of the skin. The sample with a wire diameter of 0.5 mm (transition temperature 40 °C), spring diameter of 2.5 mm, 55 turns, and initial length of 50 mm was chosen for the actuator as it achieved all the desired requirements, i.e., providing the target force, being thinnest, and having the lowest transition temperature. Upon application of a 1 kg load, the fabricated actuator was able to contract (releasing 40% of its initial length) with 0.5 Hz actuation frequency. Thus, it is capable of supporting low-frequency ADLs. The time to heat the coil to 70 °C was 1 s, and the polymer reached the temperature in 6 s. The time to cool the SMA was less than 1 s.

The designed actuator was able to provide the four target motions of the wrist, i.e., flexion, extension, ulnar deviation, and radial deviation. Five muscle-like actuators were attached at various positions with reference to the anatomical placement of tendons and joints [102].

### 4.2. Lower Limb

**Figure 5:**
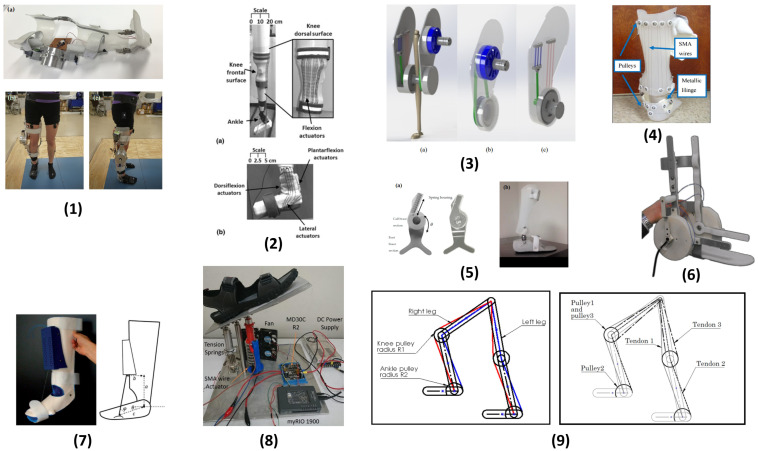
(**1**) SMA-driven knee module in Knee–Ankle Foot Orthosis [105], (**2**) Active Soft Orthotic (ASO) for knee and ankle joints using SMA wire [106], (**3**) Hybrid-Driven Knee Orthosis (KO) with SMA actuator: (**a**) Orthosis, (**b**) DC motor module (**c**) SMA spring module [107], (**4**) SMA Pulley-driven hinged Active Ankle Foot Orthosis (AFO) [108], (**5**) superelastic hinge AFO (HAFO): (**a**) Design, (**b**) Prototype [109], (**6**) Lower-Limb Exerciser with Intelligent Alloy (Leia) [110], (**7**) SHADE: SMA wire-driven ankle rehabilitation device [111], (**8**) platform-type ankle rehabilitation robot [112], (**9**) tendon-driven exoskeleton [113].

The relevant literature that reported SMA-based actuation techniques used in lower-limb exoskeletons is briefly discussed in this section and presented in Table 3 and Figure 5, which displays the target organ, target motion, actuation mechanism, cooling mechanism, and challenges.

To reduce energy consumption in SMA-based lower-limb exoskeletons without sacrificing performance, several strategies can be employed. One approach involves optimizing the design of the actuator systems to minimize weight and maximize efficiency [39]. For instance, the use of lightweight materials and streamlined designs can significantly decrease the energy required for actuation, thereby enhancing the overall efficiency of the exoskeleton.

Additionally, implementing feedback control systems that monitor user movements and adjust actuator responses accordingly can lead to more efficient energy use. By tailoring the actuation to the specific needs of the user, these systems can reduce unnecessary energy expenditure while maintaining effective support during rehabilitation [10].

#### 4.2.1. SMA-Driven Knee Module in Knee–Ankle Foot Orthosis (KAFO)

Based on the superelastic properties of Nitinol, a Nitinol-based knee actuation system for KAFO (Figure 5, (1)) was proposed to provide knee extension and flexion. The design involved the integration of a dynamic actuator comprising two components, one for the stance phase and the other for the swing phases. Each component consisted of a parallel combination of a superelastic torsional rod (with a diameter of 3.43) and a torsional spring. These two components were housed within the outer layer of the dynamic knee joint. The functionality of the actuator was tested and confirmed with a typical knee stiffness. This knee actuator was mounted on a conventional passive KAFO and then evaluated through several motion analyses on healthy subjects. It was found that the knee-actuated KAFO and slow walking had very close stiffness profiles, making the knee-actuated KAFO suitable for slow walking applications [105,114,115].

#### 4.2.2. Active Soft Orthotic (ASO) for the Knee and Ankle Joints Using SMA Wire

To develop an SMA actuator (Figure 5, (2)), 0.25 mm diameter SMA wire was wound into springs with an internal diameter of 0.51 mm after annealing at 400 °C. The actuator consists of four lines of SMA coils. A fixed current of 500 mA was used to trigger the phase transition to the austenite state by the Joule Heating Effect. The ASO comprised four sets of these SMA actuators each on the dorsal and frontal side of the knee to assist with flexion and extension, respectively. The unstretched length of these actuators was 17.5 cm for both the front and back legs. The ankle ASO comprised four sets of SMA actuators, with one set on the frontal surface, one set on the dorsal surface, and one set on the lateral surface to assist with dorsiflexion, plantarflexion, and pronation–supination, respectively. A silicone coating was applied to the top and bottom of the orthotic to prevent slippage on the leg.

The orthotic was controlled in a binary fashion by passing current through the springs to trigger a phase transformation through Joule Heating. When all four actuators of the knee were actuated, they provided a steady-state deflection of 34°, which gave a significant assist during the swing phase. This was achieved in a response time of 25 s, which is not suitable for immediate gait assistance. It was observed that shifting from one activated actuator to two would triple the response rate and provide an additional 15.7° of deflection. When shifting from two activated actuators to three, the response rate increased by 38% and an additional 4.1° of deflection was achieved. However, upon shifting to four activated actuators, the response rate remained the same and only 3.1° of additional deflection was achieved. The additional flexion provided by the ASO was found to decrease with an increase in the initial knee angle. A maximum voltage of 18 V was provided, yielding a current of 0.4 A for each of the four actuators, leaving the total power consumption at 28.8 W. This means that for continuous operation of the ASO and providing assistance to both the knee and ankle, the battery life would be very limited. This can be remedied by increasing the maximum voltage to obtain a higher current for a faster response time, but this would impact consumer safety. The cooling time for the ASO was 40–50 s due to the absence of a forced convection cooling mechanism being present [106].

#### 4.2.3. Hybrid-Driven Knee Orthosis (KO) with SMA Actuator

A Hybrid-Driven Knee Orthosis (Figure 5, (3)) was developed, utilizing a combination of a DC motor module and an SMA spring actuator module. The SMA actuator module consists of two antagonistically arranged SMA actuators, each equipped with three SMA springs in the same initial state. These actuators are interconnected and fixed to the knee joint shaft pulley using a belt. Upon activation of the SMA actuator, one of the actuators undergoes stretching while the other remains in an unstretched state. By heating the SMA actuator to induce martensitic transformation, the spring contracts along its axis, resulting in the rotation of the knee joint through the pulley. The torque generated on the pulley output shaft is transmitted to the rotation shaft of the knee joint through a set of planetary acceleration gears. As the actuator returns to its initial state, the other actuator is stretched. Both sides of the SMA actuators are heated to facilitate spring extension and contraction, allowing for the corresponding knee flexion and extension.

To address the limitation of small and inaccurate output torque from the SMA driver, a DC motor module was integrated into the design. The DC motor module and SMA module are symmetrically arranged on both sides of the lower limb [107].

The geometric parameters of SMA springs are given in Table 4:

A single SMA spring can provide a maximum force of 0.25 N [107]. Therefore, the maximum force exerted by three SMA actuators in parallel is 0.75 N. The main advantage of this design is that a large torque can be provided to the knee joint with a low magnitude force [107].

#### 4.2.4. SMA Pulley-Driven Hinged Active Ankle Foot Orthosis (AAFO)

An Active Ankle Foot Orthosis (AAFO) (Figure 5, (4)) was developed using Shape Memory Alloy (SMA) wires. The AAFO incorporated a total of fourteen plastic pulleys, which were securely attached to the brace using screws and spacers, having an eight-wire configuration in a parallel combination. The length of the SMA wire was selected such that strain recovery could be completed in one cycle. With 4% strain recovery and a 25° total angular variation, the required length of the wire was 90 inches.

This AFO was intended to be used in the treatment of drop foot. Therefore, it only catered to the dorsiflexion motion of the ankle. The wire radius chosen for the SMA wire was 0.01′ or 0.254 mm. The SMA wire provided a force of 70 N to overcome an ankle moment of 2.1 Nm. The SMA wire was activated using a power supply (1 A 35 V). The patient showed keenness in participating in further trials of the prototype because he did not experience any discomfort during its use [108].

#### 4.2.5. Superelastic Hinge AFO (HAFO)

An AFO (Ankle Foot Orthosis) device (Figure 5, (5)) was designed, comprising a brace consisting of two parts, two NiTi springs, and two hinges equipped with an internal mounting hole for attaching the springs.

Compared to a stainless-steel spring, using a NiTi spring in the AFO can provide a more normal range of motion to the ankle. The NiTi spring-actuated AFO was found to have a stiffness profile very close to that for normal walking.

The springs used were made from a 1.07 mm diameter NiTi wire. This allowed for a higher plantarflexion angle (about 2.5°) compared to that provided with the steel springs. It was confirmed that NiTi springs can provide both plantar flexion and dorsiflexion close to that for a walking situation. For NiTi springs, GRF drops as normal walking due to more ankle flexion being provided. The proposed HAFO provides a high ROM (7.9°) and increased joint ankle moment (0.55 Nm/kg).

The device had a few limitations. Firstly, it was designed to only facilitate someone weighing 72 kg and is unsuitable for anyone weighing above that. Secondly, the device was tested on only one subject. This provided a very limited assessment of the device. Thirdly, only GRF was used to study the moments, which does not give muscle activity evaluation data [109].

#### 4.2.6. Lower Limb Exerciser with Intelligent Alloys (Leia)

Leia (Figure 5, (6)) was designed for the treatment of foot drop. It consists of a pair of NiTi-based solid state motors mounted on an orthosis. These actuators contain trained NiTi wires of a sufficient length capable of providing 40° of motion around the ankle axis, providing a maximal torque of 100 Ncm. This was achieved by coiling the wire around a spiraling sequence of pulleys within the actuator housing. The actuator was designed such that no portion of the wire was deformed above 40% during actuation. A 0.25 mm diameter wire was used in a doubled arrangement to provide a parallel pull of two equal and perfectly synchronized fibers. This is performed in order to provide a sufficient torque with reasonable actuator dimensions and a thin wire.

Leia comprises a hinged aluminium structure above and below the ankle joint, to allow the constant alignment of the axis of rotation of both actuators and the axis of dorsiflexion. It is strapped to the posterior part of the leg using Velcro straps, while the sole of the foot is placed on the distal part of the orthosis. This arrangement provides the appropriate interface for the transfer of torque to the patient’s ankle. On either side of the hinge, two rotary actuators allow for movement of the foot relative to the leg, to allow ankle dorsiflexion. Leia was activated for a full stroke using 7 s pulses of a direct current, 0.7 A (per actuator), with 30 V DC. It was controlled by a surface electromyography (sEMG) signal from the tibialis anterior muscle [110].

#### 4.2.7. SHADE: SMA Wire-Driven Ankle Rehabilitation Device

An SMA-driven ankle rehabilitation device (SHADE) (Figure 5, (7)) was designed for the treatment of foot drop, using two thermoplastic shells that were lined with soft foam. The shells were strapped onto the frontal aspect of the shin and the foot using Velcro bands such that the hinge and the ankle aligned perfectly. Planar hinges were used to limit abduction, adduction, eversion, and inversion, thus making it unsuitable for patients with severe malformations out of the sagittal plane. With this configuration, actuation pull is transferred through inextensible threads through two linear actuators that are fixed on the front of the leg shell.

The linear stroke and the output force are dependent on the fixation points of the actuators and the distal ends of the inextensible threads through the lever arm. The actuators only produce dorsiflexion, while plantarflexion is produced by gravity and viscoelastic effects.

A DC generator was used to power the system, which was not present on the leg shell itself for device stability and to limit the weight. To control this device, an open-loop control system was used for passive exercises while a closed-loop control system was used for assistive rehabilitation, taking an electromyographic signal from the tibialis anterior as an input. An angular movement between the range of −5° to +10° could only be achieved with a linear stroke of 2.5 cm. The length of the wire must be 83 cm, considering a deformation of 3%. Having a free-standing wire of that length is impractical, hence, a compact actuator is needed. For this purpose, the SMA wire was coiled, which was also responsible for local deformations. The actuator comprised a cartridge containing the SMA wire, wrapped around two arrays of ten pulleys, fixed at one end to the inextensible thread and the other end fixed to the housing. A pseudoelastic spring with a negligible pull was used to keep the NiTi wire taut.

It was found that a maximum load of 13 N could be applied on each actuator with a maximum torque of 200 Ncm. If a NiTi wire of 250 µm diameter is selected, it can achieve maximum stress of 266 MPa. For plantarflexion, full martensite detwinning is required. With a resisting torque between 150 and 450 Nm and geometric parameters, martensite detwinning is always satisfied between the required range of angular movement, −5° to +10°. If a diameter of 12 mm is assumed for the pulleys, a localized strain of 2% is achieved. Due to the maximal deformation limit of 3%, only 1% of strain is actually available for actuation and can be used to calculate the length of wire to be coiled within each actuator. Within the 3% strain and 300 MPa stress limit, a 30–100k life cycle can be expected [111].

#### 4.2.8. Platform-Type Ankle Rehabilitation Robot

An SMA-based platform-type ankle rehabilitation robot (ARR) (Figure 5, (8)) provided dorsiflexion and plantarflexion motion at a range of 30° with a 3 kg maximum payload, which is the maximum weight of the foot with a safety factor of 1.5. The foot platform was fabricated using an aluminium plate to specifically cater to the Malaysian population, so the average foot size and weight there were taken as a reference. The center pillar was placed below the ankle to minimize knee motion and kinematic incompatibility between the foot and the robot. A detachable sandal was added above the foot platform to allow the physician to properly position the foot.

The design most suitable to supplementing the displacement provided by the SMA wire was chosen for the actuator. The design also allowed for flexibility in terms of increasing or decreasing the stiffness of the actuator by either changing the number of SMA wires or changing the diameter of the wire. This actuator was also capable of working in tandem with other actuators or elements.

The actuator is composed of various components, including metal plates, an aluminum cylinder, a rubber ring, an aluminum connector, and a plastic shaft. Aluminum was selected as the material for the intermediate metal plates due to its conducive properties. It enables the SMA wire to effortlessly pull the plates and serves as a heat sink for rapid cooling, aiding in the expansion of the SMA wire. The bottom metal plate is firmly attached to the central plastic shaft, while the top metal plate is connected to the aluminum cylinder, which, in turn, is connected to the foot plate.

This configuration enables relative motion between the metal plates, while a rubber ring serves as a thermal insulator, preventing heat transfer to other components of the actuator. The SMA wire is coiled around eight metal plates, weaving from one notch on the top plate to another on an adjacent plate, until it reaches the stationary metal plate at the bottom. From there, the wire is looped around the stationary plate and back to the starting point. This weaving process is repeated twelve times, creating a counter-helical rotary pattern with the twelve parallel SMA wire formation using a single length of wire. The weave is then secured to the top plate, along with the other end of the wire. This arrangement allows a 3 m wire with a diameter of 250 µm to achieve a stroke length of 5 cm. The maximum force exerted by this actuator is 110 N.

To heat the SMA wire, the Joule Heating Effect is utilized to reach the austenite finish temperature. To facilitate the wire’s cooling process back to the martensite start temperature, four conventional on–off fans are employed. Through periodic forced convection cooling, the SMA wire is able to cool in 3.57 s, compared to the 15.32 s it would take under natural convection. This setup results in a cycle time of 5.71 s, while maintaining consistent actuation timing throughout the trials [112].

#### 4.2.9. Tendon-Driven Exoskeleton

A conceptual design for a tendon-driven exoskeleton (Figure 5, (9)) was suggested for climbing stairs. It was meant to provide 50% assistance to the intended, the elderly. In the initial proposed design, two tendons were used for each leg, one for the ankle and another for the knee. The knee and ankle joint provided assistive torque through pulleys, operated by tendon tension force.

A major issue with this design was that when the user decided to bend their knee, the force would resist the stretching force or the cooling deformation force of the wires, as one part of the loading cycle stretches when it cools. Hence, another tendon is needed for each joint to produce a torque in the opposite direction to counteract this phenomenon.

The diameter of the SMA wire was based on a compromise between the pulling force and the reaction time. Based on calculations, a wire diameter of 0.38 mm was chosen, which would cool in 10 s, producing a heat pull force of 22.5 N and a cooling deformation force of 9 N. Bundles of 160 wires were used for tendon 1, 65 wires for tendon 3, and 70 wires for tendon 2.

The rest time, the cooling time for the SMA wire, takes about 90% of the climbing cycle. To reduce this, forced convection would be a requirement. Since the intended users were the elderly, this was not considered an issue. An appropriate space is required between the wires for faster cooling, which was excluded from the design because the wires were mounted in a single row.

The major challenge for the system was connecting the wire in an optimal manner while allowing easy cooling to have good mechanical performance. Another challenge is that the energy consumption is a hundred times more than an electric motor. To make the system portable, a heavy battery would be required, which would make the system less feasible. The two main advantages found for this system were high energy efficiency and low response time. However, this system is only practical for climbing a flight of 100 stairs [113].

## 5. Applications of SMAs in Smart Textiles

New textiles having the ability to adapt, sense, and react can be developed because of the emergence of high-performance fibers and smart textiles. The modification of Shape Memory Alloys has allowed for their incorporation into woven structures. The production of hybrid blends has now become possible through yarn spinning. This has provided unique woven textile actuators that react to temperature change through deformation [116].

To achieve scalable and three-dimensional actuation, active material fibers are being used to create smart textile actuators through traditional processes such as knitting [117], embroidering [118], and weaving [119]. Although carbon nanotubes [120] and shape memory polymers [119] have been investigated, SMA knitted actuators garner special interest because they can provide greater actuation contractions (>40%) while working with loads in the magnitude of 1–100 N [121].

Examples of active textile materials using SMAs are textile-based wearable devices using active SMA knits for self-fitting [122] and active compression [123,124], discrete SMA fiber active compression garments [125,126], SMAs incorporated in a felted textile used to make shape-changing garments for visual communication [127], and active orthoses containing SMAs patterned into a textile substrate [106].

To use SMAs in yarn production, it is important to provide heat treatment to the material because the conventional yarns that are the major elements in yarn formation are not able to endure high temperature. Heat treatment is important for the SMA to remember its prescribed shape. This process requires a series of heating and quenching actions.

Special spinners with custom configurations are needed to achieve yarn formation using SMA wires. At lower twist levels, the SMA can cause certain obstructions in the production of yarn formation with adequate dimensional stability. This is because of the surface quality of the alloy, which prevents other yarns form adhering to its surface. This makes the alloy unable to sustain its position in the core and intermittently protrude outside the yarn structure. To achieve good dimensional stability, overfeeding is required in at least one of the rollers. Overfeeding is achieved by increasing the wraps per minute (WPM), and it produces a textured surface that has a bulky yarn formation. This is performed in order to allow the SMA to move the yarn formation into a coil shape upon stimulation [116].

The garter knit pattern with alternating rows of forward (knit) and backward (purl) loops was investigated for linear contractile actuation deformations. During the knit manufacturing process, the SMA filament is bent to form a network of interlocking adjacent knitted loops. When thermally activated, the increase in the volume fraction of austenite results in a larger SMA fiber bending stiffness and causes partial recovery of the bending deformations that had been imposed [128]. A dimensionless parameter, knit index ($i_{k}$) [129], is used to describe the mesoscopic loop geometry of the ideal knitted loops. The loop geometry is completely dependent on the loop length [130]. The knit index relates to the filament diameter (*d*) and the void area enclosed by the knitted loop ($A_{l}$) through the following equation:

(1) $$i_{k}=\frac{A_{l}}{d^{2}}$$

The SMA knitted actuator dimensions are described through two macroscopic geometric properties, the number of loops in the horizontal (g) and the vertical (h) directions. If a knitted actuator has loops with a constant knit index, it is called homogeneous. Otherwise, it is called inhomogeneous [128].

In contractile SMA knitted actuators, the %-actuation contraction (ε) relates to the high-temperature partially austenitic knit length ($l_{a}$) and the low-temperature fully martensitic knit length ($l_{m}$) through the following equation:

(2) $$\varepsilon =1-\frac{l_{a}}{l_{m}}$$

A large external load and a lower knit index induce high average stress within an SMA knitted actuator, increasing both actuation and relaxation temperatures.

### Recent Advancements in Smart Clothing Using SMA-Based Actuators

Smart clothing used SMA wire actuators to provide assistive moments and forces to the wearer’s ankle. The SMA wire actuator could be easily embedded in a textile without extruding parts. The SMA wire used was 0.006 inches in diameter, which was deemed suitable to provide fast power for gait assistance. The clothing had a mass of 428.5 g without a power battery and could generate an ankle moment of 100 Ncm in each ankle during the gait cycle with a maximum moment at an actuation current of 0.8 A. It had an activation time of 0.5 s, the duration for the heel-off phase during walking [43].

A multifunctional elbow brace (MFEB) was developed using a knitted SMA fabric. The MFEB was capable of knitting modules having different actuation characteristics through the simultaneous application of heat and pressure in a hand-knitted fabric containing SMA wires (diameter = 0.2 mm). A current of 0.3 A (14 V) actuated the SMA. At the deformation temperature of 70 °C, the SMA showed a shrinkage in the axial direction of 4–5%. A pressure of 0.2 kPa and a temperature of 40 °C for 10 min improves blood flow by 8.49%. It was also able to increase the ROM of the elbow by 2.4° [131].

A spring-based fabric muscle (SFM) was developed using SMA to provide high force and contractility. The SFM used bundled SMA springs with proven performance. The SFM used 20 SMA springs (wire diameter = 0.5 mm, spring diameter = 2.5 mm, 52 turns) with five springs in each of the four layers, connected in series. The SFM had a combined resistance of 1.44 Ω and required a current of 10.2 A and a voltage of 14.69 V for activation. Upon heating to a temperature of 70 °C, the SFM contracts, showing a contraction strain of 50%, generating a force of 10 N or greater. Without load, it could generate a maximum contraction stress of 67% [132].

A Suit-Type Wearable Robot (STWR) was fabricated using an SMA-based fabric muscle (SFM) to assist the muscular strength of the wearer and could be comfortably worn anywhere and at any time, even with no power supply. The STWR had a simple structure and weighed less than 1 kg. The STWR comprised SFMs, wire encoders to measure the contraction length of the SFMs, and BOAs to fix the actuators on the forearms. To keep the STWR in place, a shoulder strap was attached inside the STWR, and a 100 mm long strap was additionally sewn and attached to the shoulder strap. To make the SFM easily replaceable, only one end of the SFM was fixed to the tip of the attached strap. The other end of the SFM was fixed to the lower sleeve of the garment using wires. A mannequin with wooden arms (with artificial skin made from Ecoflex 00-30, Smooth-On, Inc., Macungie, PA, USA) wearing the STWR was used for experimentation. For the experiments, the step–response performance for lifting a 2 kg barbell and a 4 kg barbell was noted. The position of the arm was changed in 10 mm intervals through actuation. It was found that when lifting a 2 kg barbell, the target position was achieved in less than 1 s except for in the 0–10 mm step. The response speed was much slower for the 4 kg barbell [133].

A pneumatic–SMA hybrid soft exoskeleton was designed that integrates strain sensors and the two types of soft actuators, an SMA and a pneumatic bladder. It consisted of an SMA actuator harness, sensing shirt, and the bladder housing unit. The shirt contained 14 strain sensors distributed over the shoulder joint. The SMAs were used to create linear forces or displacements to move the arm in the horizontal plane, while the pneumatic bladders were inflated to lift the arm through the change in shape as well as volume. For the SMA actuator, the SMA wires (diameter = 0.304 mm, activation temperature = 70 °C) were formed into springs (outer diameter = 1.219 mm). To form their shape, they were heated to 450 °C for 10 min. To avoid manual resetting of the SMA wires, the SMAs were housed in fabric braids that provide the antagonistic elastic force for SMA activation and provide faster relaxation when the SMA is not powered [134].

To provide a therapeutic compression treatment for orthostatic hypotension, a stocking was designed using SMA wires (diameter = 8 mm). The stocking was made of three panels crocheted together. Each panel contains SMA wire and Kevlar aramid fiber yarn. The panels were each able to provide different pressures by changing the ratio of active SMA wires to passive aramid yarns as well as the stitch size. High-pressure panels were placed closer to the ankle, and low-pressure panels were placed further up the leg, to promote venous return. If the number of SMA wires was kept greater than the aramid yarns, the panel was able to provide higher pressure and vice versa [123].

The relevant literature reporting fabric-based SMA rehabilitation devices is presented in Table 5, which shows the target organ, target motion, actuation mechanism, cooling mechanism, and challenges.

## 6. Conclusions

Due to their ‘soft’ nature, the use of SMA-based actuators in rehabilitative exoskeletons for injuries and neuromuscular conditions is favorable. The key challenges with using SMA-based actuators are their low working frequency, low energy efficiency, and hysteresis. These problems stem from the fact that SMAs need a significant amount of heat for actuation, have low cooling rates, and have low strain recovery during cyclic loading. Many strategies have been proposed and implemented to solve these challenges to a variable degree of success. To design an effective actuator, it is important to consider its intended application, including its ergonomic features, the target organ, and joint. Kinematic analysis of the target joint gives the force and torque requirements. The design should fulfill the following requirements:

- Light weight, small size, easy to attach, and simple structure.
- Main degrees of freedom (DOF) for each joint to be supported.
- To accommodate sudden failures, a safety mechanism must be in place.
- The target organ to be able to freely interact with the environment.

It has also become essential to simulate the interaction of the exoskeleton with human tissue to understand the response of the tissue to the loads applied by the exoskeleton while worn for an extended period. This allows for the correct selection of material for manufacturing the exoskeleton. The wire diameter considered in the actuator depends on the force requirement. The greater the force requirement, the larger the diameter of the wire used. Using a larger-diameter SMA wire increases the cooling time and therefore the work frequency. Therefore, different configurations have been implemented that use a small-diameter SMA wire and provide a large actuation force. Most techniques investigated rely on natural convection for cooling due to the high-power requirement and low work efficiency brought using the forced convection technique. An alternative to this is the use of periodic cooling, which reduces the power requirement to a significant extent.

The increasing use of SMA wires to make smart fabrics shows the bright prospects of SMA technology in rehabilitation. The only challenge with using SMAs in smart fabrics is that SMAs require a significant amount of heat for activation, which can only be provided by an external power source. This area requires the attention of researchers with an interest in the field.

## Figures and Tables

**Table 1:** Properties of NiTi wires of different diameters [37].

Wire Diameter (mm)	Resistance (Ω/m)	Activation Current (A)	Force (N)	Cooling Time 70 °C (s)	Cooling Time 90 °C (s)
0.15	55.00	0.41	3.15	2.00	1.70
0.20	29.00	0.66	5.59	3.20	2.70
0.25	18.50	1.05	8.74	5.40	4.50
0.31	12.20	1.50	12.55	8.10	6.80
0.38	8.30	2.25	22.06	10.50	8.80
0.51	4.30	4.00	34.91	16.80	14.00

**Table 2:** Upper-limb SMA-based rehabilitation devices.

Title	Target Organ	Target Motion/Application	Actuation Mechanism	Cooling Mechanism	Challenges
Forearm and hand rehabilitation exoskeleton with 3 degrees of freedom (DOF) [97]	Forearm and hand	Wrist flexion–extension and abduction–adduction	SMA wires actuating tendons in spring bias and differential configuration	Miniature fans embedded in the design	Restricted movement provided Noisy (due to fans) No clinical validation Limited customizability
EDGES SMA-based actuator [98]	Elbow	Spastic elbow relaxation	Polycentric hinges controlled by SMA wires	Natural convection	Limited clinical validation Slow response time
Bowden cable-based SMA actuator [99]	Elbow	Elbow flexion–extension	SMA wire actuation with the Bowden tube acting as a flexible heat dissipater	Natural convection aided by a Bowden tube acting as a heat sink	Rigid structure Limited effectiveness in treatment
Tube-encased SMA coil wrist actuator [100]	Wrist	Wrist flexion–extension and ulnar–radial deviation	SMA coil enclosed in a stretchable polymer (Ecoflex 00-30) tube	Coolant circulation system	Dislocation of wearable parts Possible misalignment of actuators
Four-bar link mechanism-based SMA finger actuator [101]	Fingers	Finger abduction–adduction and extension–flexion	Four-bar link mechanism	Natural convection	No clinical validation Rigid structure Delayed response (friction in mechanical components and use of natural convection for cooling)
ASR glove for hand rehabilitation [102]	Fingers	Finger abduction–adduction and extension–flexion	SMA wires actuating tendons connected to a platform on the forearm	Natural convection	Air fans reduce the applicability of the device Slow response time

**Table 3:** Lower-limb SMA-based rehabilitation devices.

Title	Target Organ	Target Motion/Application	Actuation Mechanism	Cooling Mechanism	Challenges
SMA-driven knee module in Knee–Ankle Foot Orthosis [105]	Knee	Knee extension–flexion	Parallel combination of a superelastic torsional rod and a torsional spring	Natural convection	Rigid structure No clinical validation
Active Soft Orthotic (ASO) for the knee and ankle joints using SMA wire [106]	Knee and ankle	Ankle dorsiflexion–plantarflexion and knee extension–flexion	Four lines of SMA coils	Natural convection	Slow response time
Hybrid-Driven Knee Orthosis (KO) with SMA actuator [107]	Knee	Knee extension–flexion	Two antagonistic actuators connected and fixed on a knee joint shaft pulley by belt	Natural convection	Drive system is bulky High complexity of control system
SMA Pulley-driven hinged Active Ankle Foot Orthosis (AAFO) [108]	Ankle	Ankle dorsiflexion–plantarflexion	Three SMA springs transferring torque through pulleys	Natural convection	Slow response time Power losses in pulleys
Superelastic hinge AFO (HAFO) [109]	Ankle	Ankle dorsiflexion–plantarflexion	Superelastic hinge for rotary using NiTi spring	Natural convection	Designed for a specific user weight (72 kg) Insufficient muscle activity data No clinical validation
Lower-Limb Exerciser with Intelligent Alloys (Leia) [110]	Ankle	Ankle dorsiflexion–plantarflexion	Rotary actuators on either side of the hinge	Natural convection	No clinical validation Incomplete detwinning of martensite at lower torques
SHADE: SMA wire-driven ankle rehabilitation device [111]	Ankle	Ankle dorsiflexion–plantarflexion	Two linear actuators fixed between two thermoplastic shells connected at a hinge	Natural convection	Limited clinical validation An additional DC generator required
Platform-type ankle rehabilitation robot [112]	Ankle	Ankle dorsiflexion–plantarflexion	Metal plates weaved with SMA wire	Forced convection using four conventional on–off fans	Not viable for ADLs Complex system architecture
Tendon-driven exoskeleton [113]	Knee and ankle	Ankle dorsiflexion–plantarflexion and knee extension–flexion	SMA wires with pulleys	Natural convection	Low energy efficiency Limited to 100 steps

**Table 4:** Geometric parameters of the SMA springs used in KO [107].

Sr. No.	Parameter	Value
1	Average Radius	12 mm
2	SMA Spring Wire Radius	0.5 mm
3	Number of effective coils	28
4	Spring length	100 mm

**Table 5:** Fabric-based SMA rehabilitation devices.

Title	Target Organ	Target Motion/Application	Actuation Mechanism	Cooling Mechanism	Challenges
Smart clothing for ankle rehabilitation [43]	Ankle	Ankle dorsiflexion–plantarflexion	SMA wires crossing each other and anchored at two points	Natural convection	No clinical validation Slow response time (no dedicated cooling mechanism)
Multifunctional elbow brace (MFEB) [131]	Elbow	Thermal and pressure therapy	Knitted SMA wires clenching the fabric upon activation	Natural convection	Muscle mass and skin thickness were not considered The durability of the MFEB was not tested
Spring-based fabric muscle (SFM) [132]	Any	Multi-purpose	Four layers in series of five springs connected in parallel	Natural convection	Complete contraction of SFM not possible (due to insulation) Slow response time
Suit-Type Wearable Robot (STWR) [133]	Elbow	Elbow flexion–extension	Knitted SMA wires clenching the fabric upon activation	Natural convection	High power consumption Slow response time (no dedicated cooling mechanism)
Pneumatic–SMA hybrid soft exoskeleton [134]	Shoulders and arms	Shoulder abduction	SMA springs housed in fabric braids to provide antagonistic elastic force	Natural convection	Excessive weight due to pneumatic inflatable bladders Weak thermal insulation
Stocking to provide compression treatment for orthostatic hypertension [123]	Shin	Compression treatment	SMA wires and aramid fibers interwoven at different ratios	Natural convection	Only a design concept Interweaving SMA wires and aramid fibers intricately requires costly equipment
